# Should we focus on sustainable thromboprophylaxis and avoid overuse of low-molecular-weight heparins?

**DOI:** 10.1016/j.rpth.2026.106794

**Published:** 2026-06-12

**Authors:** Gerard Gurumurthy, Bingwen Eugene Fan, Juditha Gurumurthy, Giuseppe Lippi, Lara Roberts, Emmanuel J. Favaloro, Jecko Thachil

**Affiliations:** 1 The Queen Elizabeth Hospital Kings Lynn NHS Foundation Trust, Kings Lynn, UK; 2 Addenbrooke’s Hospital, Cambridge University Hospitals NHS Foundation Trust, Cambridge, UK; 3 Department of Haematology, Tan Tock Seng Hospital, Singapore; 4 Lee Kong Chian School of Medicine, Nanyang Technological University, Singapore; 5 Yong Loo Lin School of Medicine, National University of Singapore, Singapore; 6 Northern Pathology Victoria, Department of Haematology, Northern Health, Melbourne, Australia; 7 Environmental Research Group, School of Public Health, Imperial College London, London, UK; 8 Section of Clinical Biochemistry, University of Verona, Verona, Italy; 9 King’s Thrombosis Centre, Department of Haematological Medicine, King’s College Hospital NHS Foundation Trust, London UK; 10 Institute of Pharmaceutical Sciences, King’s College London, London, UK; 11 Sydney Centres for Thrombosis and Haemostasis, Research and Education Network (REN) and Institute of Clinical Pathology and Medical Research (ICPMR), Westmead Hospital, Westmead, Australia; 12 School of Medical Sciences, Faculty of Medicine and Health, University of Sydney, Westmead Hospital, Westmead, Australia; 13 School of Dentistry and Medical Sciences, Faculty of Science and Health, Charles Sturt University, Wagga Wagga, Australia; 14 Department of Haematology, Manchester University Hospitals NHS Foundation Trust, Manchester, UK

**Keywords:** low-molecular-weight-heparin, sustainable thromboprophylaxis, thromboprophylaxis

## Abstract

Low-molecular-weight heparins (LMWHs) have become a near-routine prescription for venous thromboembolism (VTE) prevention in hospitalized patients in several parts of the world. Their use may be reinforced by embedded order sets and ‘opt-out’ defaults. However, the absolute clinical benefit of universal pharmacologic thromboprophylaxis for many low-risk medical inpatients appears small. Symptomatic VTE rates are low, mortality benefits with LMWH use are not clearly demonstrated, and any reductions in thrombotic events may be offset by bleeding complications. Several analyses suggest that a substantial proportion of patients classified as ‘low-risk’ by validated risk-assessment models still receive LMWH prophylaxis in practice. LMWH also carries a largely overlooked environmental footprint because it is derived almost exclusively from porcine intestinal mucosa via resource-intensive and low-yield manufacturing processes. It is also compounded by single-use injection materials and downstream monitoring requirements. We propose that ‘sustainable thromboprophylaxis’ should be framed within appropriate, value-based healthcare. We should prioritize LMWH for patients who meaningfully benefit, reducing potentially avoidable use in low-risk settings and explicitly accounting for environmental impact as a co-consideration where clinical benefit is marginal. Achieving this requires better VTE risk stratification integrated into workflows and formal life cycle assessment to quantify the carbon costs and supply chain vulnerabilities of current high-volume anticoagulantion practice.

## 1 Introduction

Low-molecular-weight heparins (LMWHs) for thromboprophylaxis may feel like a routine prescription for some clinicians. This is because venous thromboembolism (VTE) prevention guidance is often operationalized through admission order sets, mandatory tick-boxes, and ‘opt-out’ defaults that make pharmacologic prophylaxis almost routine [1]. In one national dataset, prescribed enoxaparin increased sharply over time and reached 97,276.5 defined daily doses per thousand per year, equivalent to roughly 266,500 defined daily doses per day in outpatient care alone [2]. Another estimate is that around 100 metric tons of heparin is produced annually worldwide [3]. These numbers do not necessarily capture all inpatient prophylaxis where most unfractionated heparin/LMWH exposure occurs. It does, however, illustrate the high-volume nature of this drug class when translated into practice. Importantly, the use of thromboprophylaxis is not uniform across regions. International data suggest that in many parts of the world, thromboprophylaxis remains underused among medical inpatients and with marked geographic variation in the proportion receiving adequate prophylaxis [4]. By contrast, some highly protocolized healthcare systems have implemented mandatory VTE risk assessment, electronic prompts, audit metrics, and performance-linked incentives that may favor near-universal assessment and high prophylaxis uptake. England is the clearest national example of this model, whereas the United States has developed VTE performance measures linked to quality reporting and accreditation processes [5,6]. The scale of use therefore raises a more nuanced question, not whether thromboprophylaxis is broadly beneficial or harmful, but whether implementation in some settings has extended prophylaxis beyond those most likely to benefit.

## 2 From Underuse to Overuse of Heparin Thromboprophylaxis

In 2008, the US Surgeon General highlighted the significant issue of VTE being one of the most dangerous but avoidable hospital-acquired complications [7]. Approximately half of all VTE events occur during or after a recent hospital admission for surgery or acute medical illness [[8], [9], [10]]. Clearly, patients who are at moderate to high risk for thrombosis are going to benefit from thromboprophylaxis, which could reduce the risk of VTE. This distinction is crucial as contemporary guidelines do not advocate universal pharmacologic prophylaxis for all hospitalized medical patients but rather prophylaxis targeted to those at increased thrombotic risk and acceptable bleeding risk [11]. In this context, it has been demonstrated that offering universal pharmacologic thromboprophylaxis to all eligible medical patients is the most cost-effective strategy in terms of lifetime costs and quality adjusted life years [12]. However, medical inpatients are a heterogeneous population with varying VTE risks and thus different thrombotic thresholds. Hence, ‘universal coverage’ of these patients with pharmacologic thromboprophylaxis (with unfractionated heparin/LMWH) may not be the ideal approach compared to an individualized risk assessment model (RAM) [13,14].

The drivers behind current practice are also important (Table 1 [[14], [15], [16], [17], [18], [19], [20], [21], [22], [23]]). In England, mandatory VTE risk assessment was introduced nationally in 2010 and supported initially through Commissioning for Quality and Innovation incentives and subsequently through National Quality Requirements in the National Health Service Standard Contract. Hospitals were expected to achieve high rates of VTE risk assessment, which created a strong implementation drive for embedded assessment tools and prompts [15,16]. National analyses from England suggested that this program was associated with reductions in VTE-related secondary diagnoses and mortality [5,20,24]. In the United States, VTE prevention has also been shaped by the Joint Commission and Centers for Medicare & Medicaid Services performance measures, which includes electronic clinical quality measures that assess whether patients received prophylaxis or had documented reasons as to why no prophylaxis was given [6]. The question is whether this enthusiastic approach to thromboprophylaxis among hospitalized patients translates to significant impact clinically.

**Table 1:** Studies and guidelines exploring causes of overuse of thromboprophylaxis and any net effect clinically.

Study	Setting and design	Causes of overuse
Child et al., [15] 2013	Before and after evaluation of 2009 versus late 2010 practice in England	Showed that 2010 NICE guidance and the 2010-2011 CQUIN framework financially incentivized VTE risk assessment and secondary prevention. Highlights practical and organizational effects of reward-linked implementation.
Basey et al., [16] 2012	Mixed methods study on the effect of VTE policies in England	Found that 2010 VTE risk assessment in English hospitals was linked to financial sanctions. Identified barriers including multiple staff involvement, interruptions, lack of policy awareness, time pressure, and complexity of tools.
Galanter et al., [17] 2010	Before and after EMR implementation study in US	Pharmacologic prophylaxis at any time during admission increased from 25.9% to 36.8% after implementation of EMR. VTE on medical units fell from 0.55% to 0.33%.
Fiumara et al., [18] 2010	Electronic alert study in high-risk hospitalized patients in US	Enhanced serial alerts generated prophylaxis orders in 58.4% of patients whose physicians initially declined prophylaxis after a one-screen alert. Prior one-screen alert strategy had reduced symptomatic 90-d VTE by 41%, but many alerts were ignored.
Le et al., [19] 2017	Decision analytic model in hospitalized medical patients in US	Prophylaxis was found to be cost-effective for an average medical patient with a VTE risk >1.0%. Supports individualization by patient risk, age, and life expectancy.
Department of Health and Social Care UK [20], 2011	England national administrative dataset	Review after national guidance was implemented. Of 3.0 million adult admissions in January to March 2011, 81% were risk assessed, up from 68% in the prior quarter. The proportion rose from 47% in July 2010 to 83% in March 2011. Providers achieving >90% assessment increased from 18 in July 2010 to 115 in March 2011.
Khanna et al., [14] 2012	Single-center study of a standard admission order set in US	Implementation of a standard admission order set transiently increased VTE prophylaxis in patients who may not potentially benefit from it.
O’Connor et al., [21] 2009	Before and after study in general medical inpatients at a community hospital	Admission order sets increased the proportion of inpatients receiving prophylaxis, supporting the idea that paper or electronic order-set design itself can be a behavioral driver of thromboprophylaxis prescribing.
Bahl et al., [22] 2024	Cross-sectional study of CDS with mandatory versus voluntary VTE risk assessment in a US health system	Mandatory risk assessment within a smart order-set environment was more effective than voluntary assessment in improving risk-appropriate prophylaxis and VTE outcomes. Suggests that mandatory CDS design is a major driver of prescribing behavior.
Haller et al., [23] 2023	Quality-improvement initiative across 11 hospitals in US	Specifically designed to reduce inappropriate pharmacologic VTE prophylaxis using an EHR-based intervention that facilitated risk assessment and recommended prophylaxis for high-risk patients only. Transient improvements in reduction of overuse noted. Suggests that embedded hospital systems can drive overtreatment unless they are actively redesigned.

CDS, clinical decision support; CQUIN, Commissioning for Quality and Innovation; EHR, electronic health record; EMR, electronic medical record; NICE, National Institute for Health and Care Excellence; VTE, venous thromboembolism.

## 3 How Effective is Heparin Thromboprophylaxis?

There has been discussion on whether pharmacologic thromboprophylaxis is overused, especially in low-risk patients who stand to gain little benefit (Table 2 [8,[25], [26], [27], [28], [29], [30], [31]]). The extent of overuse varies across institutions, healthcare systems, and patient populations. This should be interpreted in the context of the underlying study design and population. Studies using validated RAMs, such as the IMPROVE or Padua risk scores, have found that up to 86% of patients classified as low VTE risk still received prophylactic heparin in some hospital cohorts [8,[25], [26], [27],^30^,^32^]. Notably, the clinical relevance of ‘low-risk’ classification remains debated. A decision analysis model suggested that pharmacologic prophylaxis becomes cost-effective at a predicted VTE risk threshold of approximately 1%, implying that even some patients labelled ‘low-risk’ by current RAMs may still fall above a treatment threshold [26]. These reports suggest that many patients with minimal thrombotic risk are given thromboprophylaxis by default.

**Table 2:** Studies describing thromboprophylaxis overuse in medical inpatients.

Study	Setting & design	Key overuse findings
Djulbegovic et al., 2021 [8]	Retrospective cohort. Two hospitals within 1 US health system with classification using EHR-based IMPROVE-VTE scoring.	69% were classified as low risk. Majority of low-risk patients (86%) received pharmacologic prophylaxis.
Grant et al., 2018 [32]	Multicenter study across 52 US hospitals. Padua-based categorization.	Mean excess use rate in low-risk patients was 79.7%. Mean excess use in high-risk patients was 32.8%. Mean underuse rate was 21.3%.
Pavon et al., 2018 [25]	Single academic center with Padua scoring from chart review.	59% were classified as low risk. Pharmacologic prophylaxis was given to 74% of low-risk patients and 71% of high-risk patients.
Spirk et al., 2015 [26]	Multicenter Swiss observational study. Geneva Risk Score used for high- versus low-risk categorization.	Among 516 low-risk patients, 245 (48%) received thromboprophylaxis. Among 962 high-risk patients, 366 (38%) received no thromboprophylaxis.
Kocher et al., 2023 [27]	Prospective cohort. Three Swiss university hospitals. Comparison of Padua, IMPROVE, simplified Geneva and original Geneva RAMs.	Depending on RAM used, 36.7% to 41.3% of high-risk patients had underuse and 37.2% to 47.6% of low-risk patients had overuse.
Creith et al., 2022 [28]	Single-center US hospital quality-improvement cohort; hospitalized medical patients. Categorization using Padua score embedded in admission order set.	Among 250 reviewed patients, prophylaxis was inappropriately given or withheld in 91 (36.4%). In low-risk patients, 58 had prophylaxis inappropriately given. In high-risk patients, 33 had it inappropriately withheld.
Repp et al., 2024 [29]	Observational analysis of pharmacoprophylaxis initiation using predicted HA-VTE risk tertiles in US.	48% of patients in the lowest predicted-risk tertile received pharmacoprophylaxis during the first 2 hospital days. 56% in the highest 2 risk tertiles did not receive pharmacoprophylaxis.
Rocha et al., 2023 [30]	Single-center observational study including both medical and surgical patients. Padua for medical patients and Caprini-based assessment for surgical patients.	Pharmacoprophylaxis was estimated to be overused in around 30.0% of patients versus 7.0% who were at risk and did not receive appropriate prophylaxis.
Flanders et al., 2014 [31]	Multicenter cohort of at-risk medical patients.	Hospitals in the highest, moderate, and lowest performance tertiles had prophylaxis rates of 85.8%, 72.6%, and 55.5%, respectively. Lower-performing hospitals did not have higher adjusted 90-day VTE hazards.

EHR, electronic health record; HA-VTE, healthcare-associated venous thromboembolism; IMPROVE, International Medical Prevention Registry on Venous Thromboembolism; RAM, risk assessment model; VTE, venous thromboembolism.

Several seminal trials have established the evidence for thromboprophylaxis [33,34]. Yet, the absolute risk reduction from prophylaxis in such groups appears to be very limited. Among general medical inpatients, the incidence of VTE without prophylaxis has been shown to be 0.5%, and heparin prophylaxis lowers this risk to 0.3% [19]. This translates to an estimated number-needed-to-treat of 450 patients to prevent 1 symptomatic VTE [19]. Moreover, clinical trials show no improvement in mortality with prophylactic LMWH, and any VTE reductions are largely in asymptomatic thrombosis [[35], [36], [37]]. In one systematic review of LMWH and related agents used for prophylaxis in hospitalized medical patients, pharmacologic prophylaxis reduced pulmonary embolism by around 3 events per 1000 patients but increased major bleeding by around 4 events per 1000 patients (an equivalent of number needed to treat of 333 and number needed to harm of 250 patients) [35]. In parallel, studies on patient preferences, adverse effects of heparin (ie, heparin-induced thrombocytopenia), and unnecessary use of resources, including nurse workflow constraints and medication costs, all further compound the need to question blanket (or indiscriminate) thromboprophylaxis [17,34].

To summarize, many patients are exposed to LMWH thromboprophylaxis, but the benefits are limited to a few. These concerns should not be interpreted as a rejection of thromboprophylaxis in appropriately selected patients. Rather, we argue for targeted use of thromboprophylaxis where those deemed low-risk are not prescribed it and those deemed high-risk receive it. More discriminative RAMs will certainly be of use to help identify those at risk of thrombosis and would truly benefit from thromboprophylaxis.

## 4 The Environmental Impact of LMWHs

The supply chain and production of LMHW are likely to carry a substantial environmental footprint [38,39]. Heparin is derived almost exclusively from porcine intestinal mucosa, which is closely tied to large-scale pig farming and slaughter. This is mostly because heparin is a complex polysaccharide and reproducing its exact structure using recombinant technology is very challenging; therefore, large-scale, clinically approved recombinant production has not yet been achieved (with the exception of fondaparinux, synthetic LMWHs remain under investigation in preclinical and clinical development). The entire manufacturing process is resource-intensive with low yield [40,41]. Approximately 1 kg of pig mucosa yields only 160 to 260 mg of crude heparin, and raising a single ‘heparin pig’ to maturity produces about 6 kg CO_2_-equivalent/kg of pig mass [41]. Even if one argues that intestinal mucosa is a by-product of pork production, rising pharmaceutical demand still drives additional processing, manufacturing, transport, and waste. For readers unfamiliar with what these figures mean in practice, a mature pig of around 120 kg, using the estimate above, would account for roughly 720 kg CO_2_-equivalent over its lifetime. Using the United States Environmental Protection Agency greenhouse gas equivalencies calculator, this is broadly comparable to driving an average petrol vehicle for approximately 1800 miles.

Importantly, no life cycle assessments (LCAs) have been undertaken to quantify the carbon footprint at various stages of production. Upstream, pig-based LCAs quantify emissions across feed production [39]. Midstream, heparin extraction and purification are multistep industrial processes that involve chemical handling and yield losses that likely carry a degree of energy footprint [42]. Downstream, prophylaxis is delivered through single-use syringes which adds material and disposal emissions. Each stage is carbon-intensive and is multiplied by the dose volume. If monitoring is needed, it necessitates blood collections and laboratory tests, which adds significant cost, resource needs, and carbon footprint. If a meaningful fraction of prophylaxis has little value for low-risk patients, then avoidable LMWH becomes environmental savings without sacrificing patient outcomes.

Supply chain fragility also has consequential environmental impacts. Heparin supply has previously been disrupted by contamination and adulteration, with major clinical consequences and renewed attention to manufacturing transparency [43]. Sustainable thromboprophylaxis therefore encompasses not only the reduction of environmental emissions but also the enhancement of system resilience.

Most crucially, as LMWH demand grows, the number of pigs needed to meet the demand is expected to be around a billion per year [41]. It is hence essential to implement all the possible measures that reduce this environmental impact by promoting more judicious use of thromboprophylaxis.

## 5 What Should Sustainable Thromboprophylaxis Look Like?

Sustainable thromboprophylaxis should be understood as a component of Appropriate and Value-Based Healthcare, in which ‘value’ is defined by outcomes that matter to patients relative to the costs of delivering them. Environmental impact is therefore most relevant where clinical benefit is marginal and practice variation is high, as avoiding low-value prophylaxis can improve care quality while also reducing avoidable emissions.

Sustainable thromboprophylaxis means targeted use of LMWH in patients who meaningfully benefit while explicitly accounting for the environmental costs of the high-volume drug, whose upstream supply is livestock-dependent [42] (Figure). Both the American Society of Hematology and the American College of Chest Physicians limit their recommendation to prescribe LMWH to acutely ill medical patients who are at high-risk of hospital-acquired VTE [10,44]. Models such as Padua and IMPROVE remain useful in clinical practice and are reflected in major guideline frameworks, as they provide a structured approach to identifying patients likely to benefit from prophylaxis. However, these models suffer from poor discrimination and variable performance across cohorts, which may reduce precision [45]. There has been interest in the development of more nuanced RAMs, clinical support decision tools, and initiatives to reduce overuse, some of which have been integrated into clinical workflows, with more judicious use of LMWH reported, without increasing adverse effects [21,22,[46], [47], [48]]. Further refinement of VTE risk stratification may reduce LMWH dependency and lead to less wastage. One area of recent interest is defining what constitutes ‘high-risk’ individuals who would benefit most from thromboprophylaxis. Another recent decision analysis again suggested that a probability threshold of around 1.0% may be a pragmatic cutoff for prophylaxis [47]. This would classify approximately 19% to 35% of medical inpatients as high risk and substantially reduce the use of LMWH in areas where universal adoption is common practice.

**Figure:**
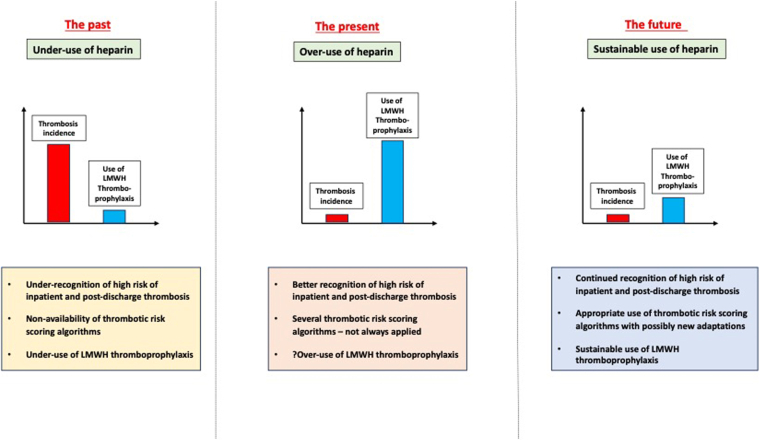
From underuse to overuse to sustainable thromboprophylaxis. LMWH, low-molecular-weight heparin.

However, better RAMs alone are unlikely to be sufficient if policy and workflow incentives continue to favor completion of prophylaxis processes more strongly than accurate discrimination of net clinical benefit. In practice, sustainable thromboprophylaxis is therefore redesigning implementation so that prescribing defaults and audit metrics are aligned with selective prophylaxis rather than use in low-risk individuals. Cross-platform clinical decision support may be particularly useful here if it is designed to promote structured reassessment and document reasons for nonprescription appropriately between RAM-defined risk and actual prescribing rather than focusing solely on assessment completion rates [[48], [49], [50]]. A further practical priority is to use existing registries and routinely collected datasets to generate further risk stratification. International registries such as IMPROVE have informed widely used thrombosis and bleeding RAMs for hospital-associated VTE [51]. These platforms could also be leveraged to assess concordance between risk and prescribing in routine practice and to determine whether de-implementation in lower-risk groups affects symptomatic VTE, bleeding, readmissions, or mortality.

Importantly, no formal anticoagulant LCA has yet to quantify the true environmental burden of LMWH across sourcing, extraction, manufacturing, packaging, transport, administration and waste disposal. As such, the environmental perspective discussed should be viewed as hypothesis-generating and as a research agenda for formal LCA. It is important to consider both an attributional estimate (allocating emissions across coproducts in the porcine supply chain) and a consequential estimate (marginal demand, ie, what happens to emissions when pharmaceutical demand changes). The ‘true’ footprint depends on the question being asked, and guideline committees and procurement groups need to understand both. Without those estimates, the debate becomes polarized between treating the material as a byproduct or as a primary production driver.

It is also reasonable to discuss alternatives to LMWH for specific contexts while being explicit that these should be treated as comparators rather than ‘low carbon’ solutions. Fondaparinux is an established option for VTE prophylaxis as supported by randomized evidence in older acutely ill medical patients [34] but has a complex manufacturing process, which is likely also carbon-intensive. Economic analyses in orthopedic settings have suggested it can be cost-saving compared with enoxaparin from the National Health Service perspective, although real-world uptake may still be shaped by renal function restrictions and acquisition cost perceptions [52]. Direct oral anticoagulants have also been investigated for use as thromboprophylaxis after orthopedic surgery [53], but their own climate footprint is increasingly quantifiable, including pathway analyses and drug-level carbon estimates [54]. Several non-anticoagulant drugs have shown efficacy in VTE prophylaxis and may have a lower carbon footprint [55]. Factor XI and XIa inhibitors are attractive in this context and have shown promising efficacy with low bleeding rates in certain contexts when compared with enoxaparin or apixaban [[56], [57], [58]]. However, these agents remain investigational or early in clinical adoption for most thromboprophylaxis settings. There are also no robust published life cycle data to indicate whether their manufacturing or use would reduce environmental burden overall.

To summarize, a pragmatic sustainable thromboprophylaxis framework should include:

(i) redesigning VTE quality metrics so that they favor appropriate targeting and reassessment rather than simple prophylaxis completion,
(ii) embedding RAM decision support that captures justified nontreatment as a marker of good practice rather than failure,
(iii) leveraging registries and linked routinely collected datasets to examine symptomatic VTE, bleeding, mortality, and prescribing intensity at a system level, and
(iv) evaluating the formal LCA of LMWH, direct oral anticoagulants, fondaparinux, and alternative agents such as factor XI inhibitors instead of assuming that oral or novel agents are necessarily lower impact.

## 6 Conclusion

LMWH will remain a mainstay of thromboprophylaxis. The argument proposed here is an overuse problem with limited benefits for many patients. Sustainable thromboprophylaxis requires a more judicious use of LMWH as demand increases alongside explicit consideration of its environmental costs. Environmental impact should be framed as a secondary co-consideration that does not supersede patient safety or clinically meaningful outcomes. Rather, it becomes most relevant in scenarios where clinical benefit is marginal, practice variation is high, or lower-impact alternatives exist without compromising efficacy. Demonstrating that optimized risk assessment reduces unnecessary LMWH use without increasing symptomatic VTE would position the climate co-benefit as a reinforcing driver of best practice implementation, rather than a distraction from clinical priorities.
